# Third ventricular glioblastoma (grade IV)

**DOI:** 10.11604/pamj.2024.49.20.44449

**Published:** 2024-09-20

**Authors:** Nikita Seth, Snehal Subrat Samal

**Affiliations:** 1 Department of Neuro Physiotherapy, Ravi Nair Physiotherapy College, Datta Meghe Institute of Higher Education and Research, Sawangi Meghe, Wardha, Maharashtra, India

**Keywords:** Glioblastoma, physical therapy, third ventricle

## Images in medicine

We are reporting a Magnetic Resonance Imaging (MRI) finding of a 57-year-old male, with an unknown history of any chronic medical illness. He presented with chief complaints of weakness over one side of the body, inability to speak, frequent episodes of vomiting and severe headache. Magnetic Resonance Imaging (MRI) findings revealed well-defined lobulated extra-axial altered signal intensity lesion noted in posterior aspect of third ventricle suggestive of grade IV Glioblastoma. It measures 3.4 x 3 x 4.2 cm indicated by red arrow (A). It appears hyperintense on T2 weighted and hypointense on T1 weighted image with mild dilatation of the bilateral lateral ventricles with mild periventricular ooze represented with blue arrow (B). Following the near total excision of glioblastoma, right ventriculoperitoneal shunting with right fronto-temporal-parietal craniectomy was performed. The patient had left-sided hemiplegia with reduced level of consciousness. For additional treatment, he was referred to a physiotherapist. He had weakness in his left side (tone grading scale grade - 0) and considerable sensorimotor impairment on physical examination. Multimodal sensory stimulation, muscle reeducation, tone facilitatory approach and positioning were a crucial part of physiotherapy rehabilitation. It has been discovered that a multidisciplinary strategy involving medical, surgical, and physical therapy improves treatment outcomes.

**Figure 1:**
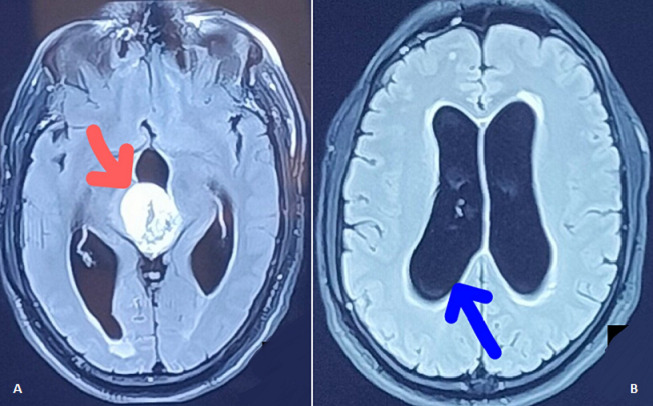
A) red arrow indicates and locates altered signal intensity lesion noted in posterior aspect of third ventricle suggestive of grade IV Glioblastoma; B) blue arrow indicates mild dilatation of the bilateral lateral ventricles with mild periventricular ooze

